# Intra-Individual Variability of Lipoprotein(a) After Acute Coronary Syndrome: A Long-Term Cohort Study

**DOI:** 10.3390/jcm15051938

**Published:** 2026-03-04

**Authors:** Nelsa González-Aguado, Jose Ignacio Larrubia-Valle, Rafael Franco-Hita, Alberto Piserra-López, Arancha Díaz-Expósito, Victoria García-Ruiz, Fernando Puyol-Ruiz, Óscar Barquero-Alegre, Fernando Carrasco Chinchilla, Antonio Domínguez-Franco, Amalio Ruiz-Salas, Jorge Rodríguez-Capitán, Alejandro Pérez-Cabeza, Mora Murri, Francisco Javier Pavon-Moron, Juan José Gómez-Doblas, Manuel Jiménez-Navarro, Francesco Costa

**Affiliations:** 1 Department of Medicine, Heart Area, Hospital Universitario Virgen de la Victoria, IBIMA Plataforma BIONAND (Instituto de Investigación Biomédica de Málaga y Plataforma en Nanomedicina), CIBERCV (Centro de Investigación Biomédica en Red Enfermedades Cardiovasculares), Universidad de Málaga (UMA), 29010 Málaga, Spain; 2 Centro de Investigación Biomédica en Red de Enfermedades Cardiovasculares (CIBERCV), 28029 Madrid, Spain; 3 Department of Biomedical and Dental Sciences and of Morphological and Functional Images, University of Messina, 98122 Messina, Italy

**Keywords:** lipoprotein(a), intra-individual variability, acute coronary syndrome, reclassification, long-term follow-up

## Abstract

**Background:** Lipoprotein(a) [Lp(a)] is a causal and independent risk factor for atherosclerotic cardiovascular disease (ASCVD) and is largely genetically determined. However, recent studies indicate significant intra-individual variability, particularly among patients with intermediate Lp(a) levels (30–50 mg/dL). Yet, data on long-term variability are limited, and acute coronary syndrome (ACS) may further influence Lp(a) levels, raising questions regarding the optimal timing of assessment after ACS. **Methods:** We studied 235 ACS patients across two follow-up cohorts. Baseline Lp(a) was measured 24 h before hospital discharge. Cohort A had follow-up measurements at 4 months and 8 months; Cohort B had them at 5 years. Clinically meaningful intra-individual variability was defined as ≥20 mg/dL or ≥25% change. **Results:** 57.9% of patients exhibited clinically significant Lp(a) variability. Changes in risk category occurred in 15.3% of patients in the baseline high-risk group, 60.6% of patients in the intermediate-risk group, and 5.5% of patients in the baseline low-risk group. In the multivariable analysis, incomplete revascularization was an independent predictor of high Lp(a) variability (odds ratio (OR) 2.22; 95% confidence interval (CI) 1.14–4.31; *p* = 0.02) while female sex and age-adjusted menopause showed a trend (OR 1.92; 95% CI 0.93–4.00; *p* = 0.08 and OR 11.18; 95% CI 0.79–157.58; *p* = 0.07, respectively) without reaching statistical significance. The median absolute changes from baseline to 4-month and from baseline to 5-year follow-up were 7.9 mg/dL (interquartile range (IQR) 3.0–18.9) and 10.7 mg/dL (IQR 3.0–21.7), respectively. Concordance between 4- and 8-month Lp(a) measurements was excellent. **Conclusions:** Early post-ACS intra-individual variability in Lp(a) is common, mainly affecting risk reclassification in intermediate-risk patients. In those patients, early, targeted, repeat Lp(a) measurement may improve cardiovascular risk stratification, whereas mid- to long-term reassessment appears unnecessary.

## 1. Introduction

Lipoprotein(a) (Lp(a)) is a molecule of low-density lipoprotein cholesterol (LDL-C) with an apolipoprotein(a) (Apo(a)) covalently bound by a disulfide bridge to apolipoprotein B-100 (apoB-100). Several mechanisms are involved in its pathogenesis, including proatherogenic, proinflammatory, and prothrombotic effects [1,2,3].

Lp(a) levels are associated with atherosclerotic cardiovascular disease (ASCVD), calcific aortic valve stenosis, peripheral artery disease, abdominal aortic aneurysm, and major adverse limb events [1,2,3,4,5,6,7,8,9]. Moreover, Lp(a) concentrations are associated with an increased risk of both all-cause and cardiovascular mortality in the general population as well as in individuals with established ASCVD [10,11].

The association between Lp(a) levels and ASCVD risk is continuous and linear, as demonstrated in an observational study of over 500,000 individuals with a median follow-up of 11.2 years, encompassing both primary and secondary prevention populations [4]. Consequently, thresholds used to define elevated Lp(a) vary across studies, reflecting the absence of a universally accepted cutoff for risk stratification: while guidelines commonly adopt a pragmatic threshold of 50 mg/dL (≈125 nmol/L), randomized cardiovascular outcome trials often use higher cutoffs (70–90 mg/dL; ≈175–225 nmol/L) to identify higher-risk populations [12,13,14,15,16,17].

Lp(a) concentrations are largely genetically determined (≈70–90%), primarily by the LPA gene encoding apo(a), and have traditionally been considered stable over time [1,2,3].

Population studies demonstrate Lp(a) variability across racial and ethnic groups, with the highest levels observed in Black individuals, followed by those of South Asian descent [2]. Lp(a) levels also significantly increase in postmenopausal women, likely related to decreased estrogen, although it remains unclear whether this reflects aging or menopause itself [18,19,20].

Yet, recent large-scale studies have reported significant intra-individual variability in approximately 20–25% of patients, with the highest reclassification rates observed in individuals with borderline Lp(a) levels (30–50 mg/dL). However, long-term data are still limited. Despite these findings, the magnitude of these fluctuations and their impact on cardiovascular adverse outcomes remain unclear [21,22,23,24,25,26,27,28,29,30].

Furthermore, intra-individual variability in Lp(a)—and the most reliable timing for its assessment to refine secondary prevention risk stratification after acute coronary syndrome (ACS)—remains poorly characterized. Recent studies have reported significant fluctuations in Lp(a) concentrations during the acute phase of ACS, underscoring both the limitations of relying on a single measurement and the importance of defining the optimal timing of assessment to improve risk stratification [31,32,33,34].

To address this evidence gap, we evaluated long-term intra-individual variability in Lp(a) and its clinical impact on reclassification across Lp(a) risk categories in two prospective cohorts of patients with ACS, and we explored predictors of clinically meaningful variability and the optimal timing for Lp(a) assessment.

## 2. Materials and Methods

### 2.1. Study Population and Design

We conducted a prospective study comprising two distinct follow-up cohorts. The first cohort was derived from the *Optimize Risk Prediction after Myocardial Infarction through Artificial Intelligence and Multidimensional Evaluation* (ORACLE) program, a prospective, observational, multicenter study that consecutively enrolls patients with high-risk ACS. The second cohort included consecutive ACS patients admitted to a tertiary-level hospital scheduled for long-term follow-up. Detailed inclusion and exclusion criteria are provided in Appendix A. In both cohorts, patients were enrolled during the index hospitalization following percutaneous coronary revascularization (PCI). Participants were recruited between August 2024 and December 2025.

In Cohort A, Lp(a) levels were measured at study inclusion and repeated at 4-month and 8-month follow-ups. In Cohort B, Lp(a) was assessed at study inclusion and repeated at 5 years of follow-up. In both cohorts, the baseline measurement was assessed 24 h before discharge. Lp(a) measurements were performed according to a standardized method at the centralized laboratory of Hospital Virgen de la Victoria (Málaga, Spain). The immunoturbidimetric assay was employed, utilizing a polyclonal antibody against apo (a) to minimize isoform bias. Results were reported in mg/dL.

Lp(a) risk categories were established in accordance with the European Atherosclerosis Society guidelines: high-risk (≥50 mg/dL), intermediate-risk (30–50 mg/dL), and low-risk (<30 mg/dL) [13,14,35]. Clinically significant Lp(a) variability was defined as an absolute change of ≥20 mg/dL or a relative change of ≥25%. Although previous studies have frequently used a lower absolute threshold (≥25 nmol/L ≈ 10 mg/dL), cut-off values are not clearly established and show considerable heterogeneity across studies, with some defining high variability only based on an absolute change ≥10 mg/dL and others incorporating a relative change ≥25% [21,22,23,24,25,26,27,28,29,30]. We therefore selected a more conservative threshold to minimize the impact of technical variability inherent to serial Lp(a) measurements, particularly in the context of long-term follow-up and potential Lp(a) fluctuations related to the acute-phase response following ACS. The absolute threshold of ≥20 mg/dL was also used by Deshotels et al. to define high Lp(a) variability in their long-term analysis, which had a median follow-up of 15 years [23]. By applying a higher absolute threshold while maintaining the relative change criterion, we aimed to identify sustained and potentially clinically meaningful changes in Lp(a) concentrations.

Demographic, clinical and analytical data were collected at baseline and during follow-up visits, according to the study protocol. Major adverse cardiovascular events (MACE) were defined as a composite outcome of cardiovascular mortality, all-cause mortality, myocardial infarction (MI), stroke, and unplanned revascularization. This study was approved by the local Institutional Review Board, and written informed consent was obtained from all patients, or their legal guardians, before study inclusion. This study was conducted according to the ethical principles of the Declaration of Helsinki.

### 2.2. Statistical Analyses

The distribution of continuous variables was assessed using the Shapiro–Wilk or Kolmogorov–Smirnov tests, complemented by visual inspection of histograms and Q–Q plots. Based on the results of these normality assessments, parametric or non-parametric statistical tests were selected as appropriate. Continuous variables were expressed as mean ± standard deviation for normally distributed variables or as median with (Q1–Q3) for non-normally distributed variables. Categorical variables were expressed as numbers (percentages).

Comparisons between groups were performed using Student’s *t*-test or one-way ANOVA for normally distributed variables, the Mann–Whitney U test or Kruskal–Wallis test for non-normally distributed variables, and the Chi-square test for categorical variables.

Absolute and relative variability between baseline and first follow-up (4 months and 5 years) was assessed using the Mann–Whitney U test. In the subgroup with paired measurements at 4 and 8 months, intra-individual variability was assessed using the Wilcoxon signed-rank test. Agreement between risk categories was assessed using weighted Cohen’s kappa, with interpretation according to conventional thresholds.

For the regression analysis, univariate analyses were first performed to identify clinical and biochemical variables associated with high Lp(a) variability. Variables with clinical relevance or a univariate *p*-value < 0.20 were considered for inclusion in multivariable logistic regression models.

Age was forced into the multivariable models due to its clinical relevance as a potential confounder. Analyses involving menopausal status were restricted to female patients. Multivariable models were constructed using logistic regression, and results were reported as odds ratios (ORs) with 95% confidence intervals (CIs).

All statistical analyses were performed using IBM SPSS Statistics 31.0.1.0 (IBM Corp., Armonk, NY, USA). A two-sided *p*-value < 0.05 was considered statistically significant.

## 3. Results

### 3.1. Baseline Characteristics

A total of 235 patients were included, 143 from Cohort A with measurements at baseline, 4 months and 8 months, and 92 from Cohort B with measures at baseline and 5-year follow-up (Table 1). Clinical characteristics within the two study cohorts are presented in Table A1 (Appendix B). The median age was 61.1 (55.0–70.6); 79.1% were men. With respect to cardiovascular risk factors, 63% had hypertension, 74% had dyslipidemia, and 35.7% had diabetes mellitus. Additionally, 21.3% of the patients had previous MI, and 18.3% had undergone further prior PCI. Median baseline Lp(a) was 34.7 mg/dL (14.8–79.0), with approximately one-third of patients (36.2%) showing elevated baseline Lp(a). Clinical characteristics of Cohort A and Cohort B and patient categorization according to baseline and follow-up Lp(a) levels are provided in Table A2 (Appendix B).

### 3.2. Intra-Individual Lipoprotein(a) Variability and Clinical Characteristics Stratified by Lp(a) Variability

Overall, 57.9% of patients had significant Lp(a) variability, exhibiting intra-individual variability ≥25% or ≥20 mg/dL. Baseline characteristics were comparable between the high and low Lp(a) variability groups (Table 2). Patients with high Lp(a) variability showed a trend toward a worse lipid profile compared with those with low variability, although none of the evaluated parameters reached statistical significance. Notably, despite similar baseline Lp(a) levels, a trend toward lower mean Lp(a) levels during follow-up was observed in the high-variability group.

Patients with high Lp(a) variability more frequently had incomplete revascularization (31.6% vs. 18.2%; *p* = 0.020) and were postmenopausal (23.5% vs. 12.1%; *p* = 0.034).

### 3.3. Transition Between Lp(a) Categories

**Figure 1:**
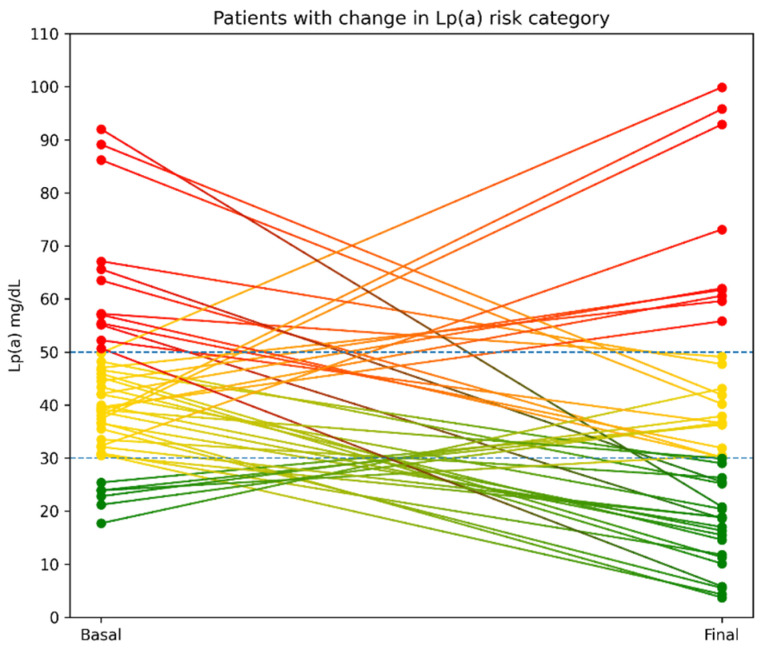
Trajectories of lipoprotein(a) from baseline to follow-up in patients who changed risk category. Each line represents an individual patient, and colors indicate the risk group at follow-up. Dashed horizontal lines represent the thresholds defining lipoprotein(a) risk categories.

The majority of patients remained in the same risk category during follow-up (81.3%). Among the 44 patients who transitioned between categories, 68.2% transitioned from a higher to a lower risk category at follow-up, whereas 31.8% transitioned from a lower to a higher risk category at follow-up (Figure 1).

**Figure 2:**
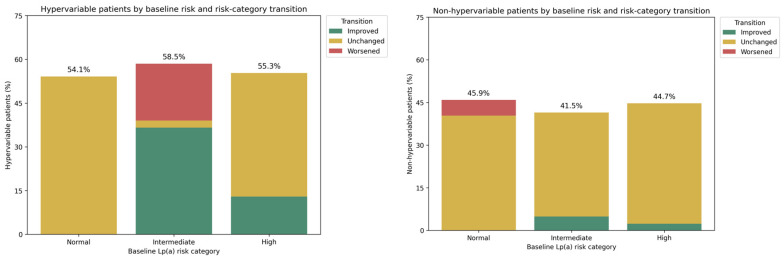
Lipoprotein(a) risk reclassification in high-variability and low-variability patients by baseline risk category.

Significant Lp(a) variability (≥25% or ≥20 mg/dL) was observed in 54.1% of patients with normal baseline Lp(a) levels, 58.5% of those with intermediate baseline Lp(a) concentrations, and 55.3% of patients with high baseline Lp(a) levels, with no statistically significant differences in variability based on the baseline status (Figure 2).

When analyzing each specific risk category in the normal Lp(a) category at baseline, 94.5% remained in the same category, and 5.5% shifted to the intermediate category, while none shifted to a high-risk category.

Among patients in the intermediate Lp(a) category at baseline, 39% remained in the same category, 41.5% shifted to the normal category, and 19.5% transitioned to the high-risk group.

Among patients in the high-risk Lp(a) category at baseline, 84.7% remained in the same category, 10.6% shifted to the intermediate-risk category, and 4.7% shifted to the low-risk category.

Detailed data on patient reclassification across Lp(a) risk categories and intra-individual variability are provided in Appendix B.

### 3.4. Multivariable Logistic Regression Analysis of Factors Independently Associated with High Lp(a) Variability

Univariable and multivariable logistic regression for factors associated with high Lp(a) variability is presented in Table 3. In multivariable logistic regression, incomplete revascularization (OR 2.22; 95% CI 1.14–4.31; *p* = 0.02) was an independent predictor of high Lp(a) variability. Female sex (OR 1.92; 95% CI 0.93–4.00; *p* = 0.08) and age-adjusted menopause (OR 11.18; 95% CI 0.79–157.58; *p* = 0.07) showed a trend toward association with high Lp(a) variability without reaching statistical significance.

### 3.5. Variability of Lipoprotein(a) According to the Timing of Determination

**Figure 3:**
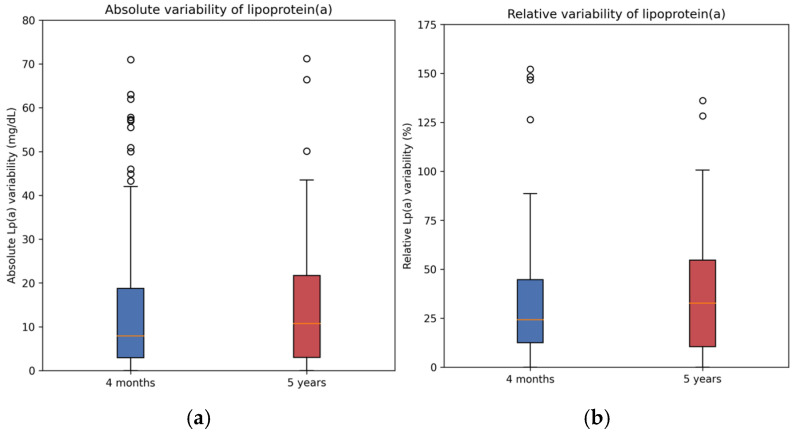
Absolute and relative variability of lipoprotein(a) at 4-month and 5-year follow-ups. Box plots show absolute (**a**) and relative (**b**) lipoprotein(a) variability. The central line indicates the median, boxes show the interquartile range, and whiskers represent the 1.5 interquartile range. Individual points denote outliers.

Lp(a) variability between baseline and follow-up measurements was compared among patients assessed at 4 months, 8 months and 5 years of follow-up (Figure 3). In Cohort A, median absolute variability from baseline to 4-month follow-up was 7.9 mg/dL (interquartile range (IQR) 3.0–18.9), whereas in Cohort B, median absolute variability from baseline to 5-year follow-up was 10.7 mg/dL (IQR 3.0–21.7). Median relative change in Lp(a) values was 25.0% (IQR 12.5–45.7) from baseline to 4-month follow-up, and 32.7% (IQR 10.5–54.6) from baseline to 5-year follow-up. No significant difference in Lp(a) values was observed between 4-month and 8-month follow-ups.

**Figure 4:**
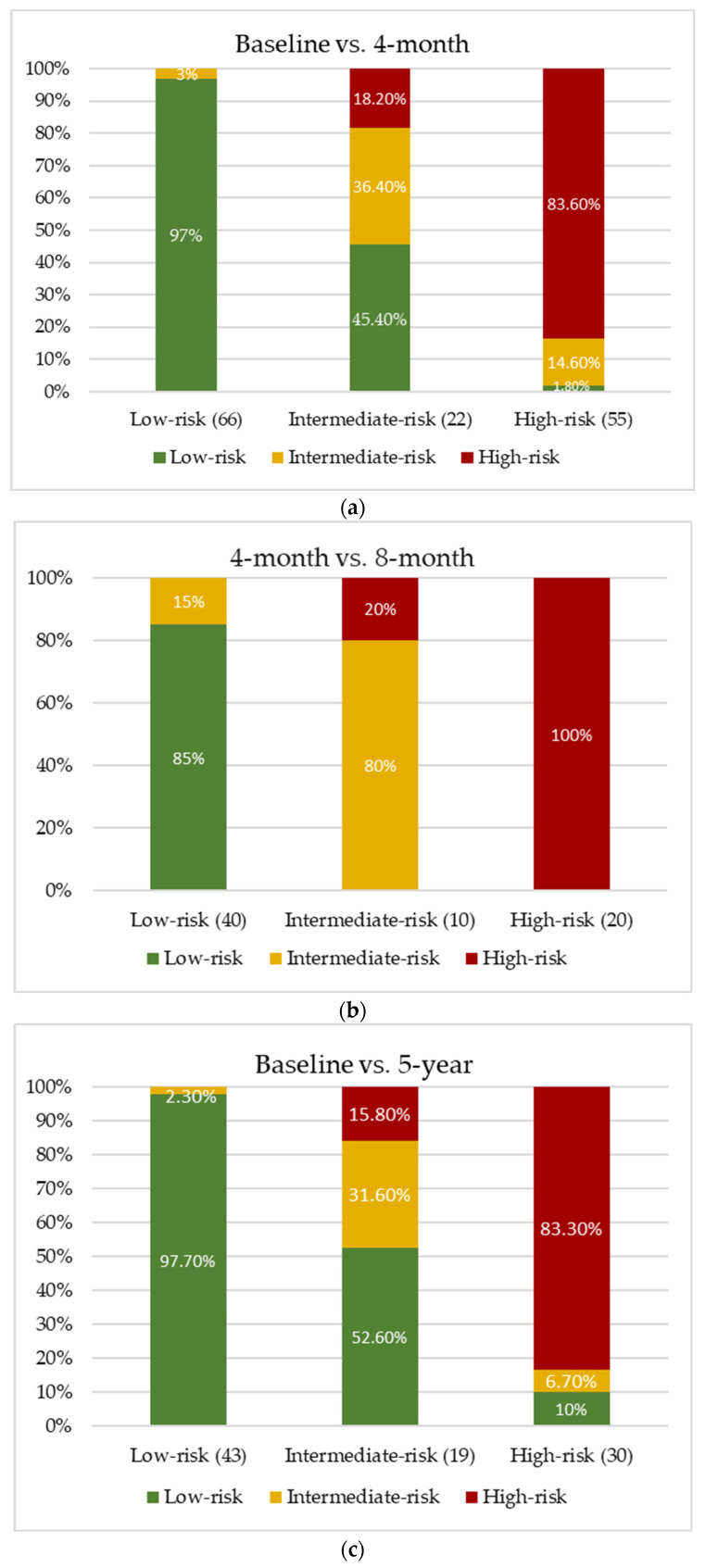
Reclassification of lipoprotein(a) risk categories across follow-up time points: (**a**) baseline vs. 4 months; (**b**) 4 months vs. 8 months; (**c**) baseline vs. 5 years. The *x*-axis represents the baseline Lp(a) risk category, and the *y*-axis shows the proportion of patients classified into each risk category at the corresponding follow-up assessment. Stacked bars display how patients shift between low-, intermediate-, and high-risk categories during follow-up.

Changes in categories among patients with repeated Lp(a) assessments at 4 months, 8 months or 5 years are presented in Figure 4. Agreement between baseline and follow-up values was strong for baseline to 4-month follow-up risk (weighted Cohen’s kappa 0.81; 95% CI 0.74–0.88; *p* < 0.001); 4-month to 8-month follow-up (weighted Cohen’s kappa 0.88; 95% CI 0.79–0.96; *p* < 0.01); and baseline to 5-year (weighted Cohen’s kappa 0.75; 95% CI 0.64–0.85; *p* < 0.01).

Reclassification figures appeared highly consistent among patients reevaluated at 4 months or longer-term follow-up (Figure 4).

## 4. Discussion

The main results of this study, which evaluated the prospective variability of Lp(a) at short- and long-term after ACS for the first time, can be summarized as follows:

- Substantial intra-individual variability in Lp(a) is common after ACS, affecting nearly six in ten patients when using clinically relevant variability thresholds. Incomplete revascularization, female sex and age-adjusted menopause were observed as potential predictors of high Lp(a) variability.
- Despite this variability, most patients remain within the same Lp(a) risk category over time, with reclassification concentrated in those with intermediate baseline Lp(a) levels, whereas patients starting in the low-risk range were essentially never reclassified at high-risk.
- Reclassification patterns were broadly comparable across reassessment time points after ACS, with similarly high category concordance and similar proportions of patients changing risk category whether Lp(a) was remeasured early or at longer-term follow-up.

Lp(a) is a lipid particle whose plasma concentrations are generally considered stable over time, largely due to its strong genetic determination [1,2,3]. However, multiple recent studies have reported clinically significant longitudinal intra-individual variability in approximately 20–25% of patients, which might affect risk classification and related prevention strategies [21,22,23,24,25,26,27,28,29,30].

In our ACS population, clinically meaningful intra-individual variability in Lp(a) was observed in 57.9% of patients; however, risk-category changes occurred predominantly among those classified as intermediate-risk at baseline, with more than half being reclassified during follow-up, whereas patients initially classified as low-risk were never reclassified.

The relatively high rate of clinically significant variability observed in our cohort (57.9%) may partly reflect the combined use of both absolute and relative changes in the definition of clinically significant Lp(a) variability. Although this approach was intended to minimize the impact of technical variability and potential transient Lp(a) fluctuations during the acute phase following ACS, the inclusion of either an absolute or a relative criterion—rather than requiring both absolute and relative changes to be met in a combined definition—may have contributed to the higher proportion of patients classified as having high variability in our study.

Studies in which a combined definition has been applied have reported a lower percentage of patients with high variability (around 20%), whereas studies using less conservative definitions have shown results similar to those observed in our cohort [21,22,23,24,25,26,27,28,29,30]. Nevertheless, heterogeneity in the thresholds used across different studies limits the standardization of variability definitions. Therefore, more homogeneous studies are needed to facilitate comparisons and to better define clinically meaningful thresholds for change.

Our findings are consistent with previous studies, conducted outside the ACS setting, in which approximately 50% of individuals with baseline intermediate Lp(a) levels were reclassified during follow-up. Deshotels et al. reported that in a cohort of 4734 outpatient individuals, 58.1% of those with baseline Lp(a) levels of 30–50 mg/dL reached levels ≥50 mg/dL after a median follow-up of 15 years [23]. Similarly, Awad et al. showed that among the 51.2% of individuals with borderline baseline Lp(a) who changed risk category, 27.9% moved to the normal-risk category, whereas 23.3% worsened to high-risk [24]. Joo et al. reported that among patients with intermediate Lp(a) levels, 22.5% transitioned to low-risk, while 29.3% progressed to high-risk [25].

These studies mostly included data from a general population in an outpatient clinic setting, while evidence in patients with established cardiovascular disease remains limited. We confirmed that, among patients included for ACS, a similar pattern in classification was present among different reevaluation timelines.

During acute events such as hospitalization for ACS, substantial metabolic and inflammatory perturbations may occur, potentially resulting in clinically meaningful changes in measured Lp(a) levels. Several interleukin-6–responsive elements within the LPA gene may contribute to fluctuations in Lp(a) concentrations during states of acute or chronic inflammation. Nevertheless, Lp(a) does not behave as a classic acute-phase reactant in ACS, as its temporal trajectory does not correlate with changes in high-sensitivity C-reactive protein (hsCRP) or other inflammatory markers [31,32,33,36,37,38]. In a prior smaller study, Ziogos et al. evaluated 108 patients with acute MI and reported an increase in Lp(a) levels at 6 months compared with measurements within 24 h of hospital admission (*p* = 0.02), with an increase >25 nmol/L observed in more than 20% of patients, which was independent of hsCRP levels [31]. Similarly, a pre-specified analysis of the randomized controlled trials *Evolocumab in Acute Coronary Syndrome* (EVACS I; ClinicalTrials.gov, NCT03515304) and *Evolocumab in Patients With STEMI* (EVACS II; ClinicalTrials.gov Identifier: NCT04082442) reported that, in placebo-treated patients, Lp(a) levels increased from measurements obtained within 24 h of hospital admission to hospital discharge and to 30 days [33]. Similar findings—suggesting a potential rise in Lp(a) from the in-hospital phase to follow-up—have been reported in other ACS cohorts, supporting consideration of repeat Lp(a) measurement to ensure accurate risk assessment [32,34].

Notably, another key finding of our study was the excellent agreement between Lp(a) risk categories at 4 and 8 months, suggesting that most intra-individual variability in Lp(a) levels after ACS occurs early, with no meaningful changes thereafter. These results, in line with prior studies, might suggest that intra-individual Lp(a) clusters during the initial phase after the acute event and remain largely stable afterwards, potentially informing the optimal timing of Lp(a) reevaluation in individuals in whom it is deemed necessary [31,32,33,34].

Our long-term follow-up cohort captured Lp(a) values after stabilization, reflecting potential true intra-individual variability rather than the transient acute-phase response.

Yet, whether risk reclassification translates into a higher risk of MACE remains to be clarified. An individual patient-data meta-analysis of statin outcome trials demonstrated that both baseline and on-statin treatment Lp(a) levels ≥ 50 mg/dL were associated with a higher incidence of cardiovascular events (hazard ratio (HR) 1.31; 95% CI 1.08–1.58 and HR 1.43; 95% CI 1.15–1.76, respectively) [39]. In contrast, Trinder et al., in a cohort of patients without coronary artery disease (CAD), did not observe statistically significant associations between Lp(a) variability and incident CAD, regardless of baseline Lp(a) levels or the magnitude and direction of change [22]. Although the clinical significance of Lp(a) variability remains uncertain, reclassification from the intermediate (30–50 mg/dL) to high-risk (≥50 mg/dL) category may have relevant therapeutic implications, as current European and American guidelines recognize Lp(a) ≥ 50 mg/dL as a cardiovascular risk modifier [13,14]. Moreover, with emerging Lp(a)-targeted therapies currently being evaluated in cardiovascular outcome trials, accurate risk categorization could become increasingly relevant for identifying candidates for future treatment. In this context, early repeat measurement in patients with intermediate baseline Lp(a) could refine risk stratification and optimize long-term preventive strategies [15,16]. Therefore, further studies are needed to assess the short- and long-term clinical impact of Lp(a) variability.

In our study, baseline characteristics were largely similar between patients with and without high Lp(a) variability, yet incomplete revascularization, female sex and age-adjusted menopause were observed as potential predictors of high Lp(a) variability during follow-up. In this setting, incomplete revascularization may represent a proxy for more diffuse and severe CAD, potentially influencing both patient risk profile and metabolic variability. In patients with incompletely revascularized CAD, residual inflammatory risk persists, partly driven by Lp(a), which carries a high burden of oxidized phospholipids that activate endothelial cells, monocytes, and macrophages, thereby promoting vascular inflammation and endothelial dysfunction [37]. Variability in Lp(a) levels may reflect fluctuations in inflammatory and thrombotic status, contributing to CAD progression and the occurrence of new ischemic events. In line with this hypothesis, large cross-sectional studies of MI patients observed that Lp(a) levels were independently associated with markers of severe CAD, including a high Gensini score (≥100), left main disease, and three-vessel disease [40].

Similarly to our cohort, systematic review and meta-analysis reported higher Lp(a) levels in postmenopausal women, in line with the finding of a potential effect of hormone replacement therapy with estrogen and progesterone in postmenopausal women [18,19,20]. Hence, our results suggest that these factors may not only affect absolute levels of Lp(a) but also its variability over time. Nevertheless, these findings should be interpreted with caution given the wide confidence intervals observed and require further confirmation in other independent cohorts.

Finally, although risk-category reclassification was common in our cohorts, it appeared to be largely an early post-ACS phenomenon. We observed excellent concordance between risk categories assigned at 4 and 8 months, indicating that once Lp(a) is reassessed after the acute phase, subsequent short-term measurements are unlikely to materially change classification. Importantly, to our knowledge this is the first study to report Lp(a) reassessment in ACS patients at long-term follow-up to 5 years, enabling estimation of the frequency and potential clinical implications of Lp(a) variability over time and informing long-term secondary prevention strategies. In this context, reclassification proportions were broadly similar regardless of whether follow-up occurred early (4–8 months) or at longer-term time points up to 5 years, suggesting limited incremental yield from routine mid- or long-term repeat testing once a stable post-ACS value has been established.

Taken together, these data support a targeted repeat Lp(a) measurement early after the index ACS event, particularly in patients with intermediate baseline Lp(a), in whom reclassification is most clinically relevant. By contrast, additional long-term repeat measurements may be unnecessary, especially in patients for whom a change in risk category is unlikely despite potential variability.

Several limitations of this study should be acknowledged. First, mid–short-term and long-term follow-up were evaluated in two separate cohorts. Although baseline median Lp(a) levels were broadly comparable between cohorts, differences in age distribution and cardiometabolic profile may have affected the stability of Lp(a) measurements over time. Therefore, direct within-patient comparisons of the magnitude of Lp(a) variability and risk-category reclassification across short- and long-term time horizons cannot be firmly established. Nevertheless, the excellent concordance observed between 4- and 8-month assessments supports the concept that most clinically relevant reclassification may occur early after ACS.

Second, generalizability may be limited considering the high-risk cohort included. The study population was predominantly male and included patients with a high prevalence of risk factors and comorbidities. Accordingly, results may not extrapolate to lower-risk cohorts.

Third, selection and attrition bias cannot be excluded, as analyses were restricted to patients with available follow-up that allowed repeated Lp(a) determinations. Nevertheless, follow-up was standardized for the overall population, and attempts for study retention were performed to reduce follow-up loss.

Fourth, long-term follow-up provides an important perspective, but a survivor bias associated with patients who remained alive at long-term follow-up cannot be excluded.

Finally, the sample size and event rate limited statistical power to robustly evaluate the prognostic impact of Lp(a) variability and reclassification on clinical outcomes and to precisely estimate associations for some candidate predictors. Further studies in larger ACS cohorts are needed to address this issue.

## 5. Conclusions

Substantial intra-individual variability in Lp(a) is common in patients after ACS. While none of the patients initially classified as low-risk was reclassified to the high-risk category, more than half of those in the intermediate-risk category experienced a clinically meaningful shift in risk classification. Incomplete revascularization, female sex and menopause emerged as potential predictors of subsequent variability in Lp(a). Moreover, our data provide no evidence that mid- to long-term reassessment offers incremental value over early post-ACS remeasurement.

Therefore, given the potential role of Lp(a) in risk stratification and its growing relevance for secondary prevention, an early, targeted repeat Lp(a) measurement may be considered in selected patients after ACS, especially if classified as intermediate-risk at baseline.

## Figures and Tables

**Table 1:** Baseline characteristics. Continuous variables are reported as mean ± standard deviation when normally distributed and as median (interquartile range) when non-normally distributed. Categorical variables are reported as n (%).

Characteristic	n = 235
Age, years	61.1 (55.0–70.6)
Male sex	186 (79.1)
Diabetes mellitus	84 (35.7)
Hypertension	148 (63.0)
Dyslipidemia	174 (74.0)
Menopausal status	44 (89.8)
Body mass index, kg/m^2^	27.9 (25.3–31.0)
Stroke	8 (3.4)
Transient ischemic attack	3 (1.3)
Peripheral artery disease	13 (5.5)
Prior MI	50 (21.3)
Prior PCI	43 (18.3)
Chronic kidney disease	118 (50.2)
Smoking status (never/former/current)	54 (23)/90 (38.3)/89 (37.9)
Alcohol consumption (never/former/current)	213 (90.6)/17 (7.2)/5 (2.1)
STEMI presentation	126 (53.6)
Number of treated lesions	2.0 (1.1)
Left main treated	15 (6.4)
Number of treated vessels	1.7 (0.8)
Incomplete revascularization	61 (26)
High-intensity statin	233 (99.1)
Ezetimibe	112 (47.7)
Total cholesterol, mg/dL	158.0 (124.5–191.0)
HDL-C, mg/dL	34.5 (31.0–40.0)
LDL-C, mg/dL	93.0 (61.8–123.3)
Non–HDL-C, mg/dL	121.0 (91.8–156.0)
Remnant-C, mg/dL	26.0 (21.0–33.0)
TG, mg/dL	139.0 (108.0–183.0)
VLDL, mg/dL	27.8 (21.6–36.6)
TC/HDL ratio	4.4 (3.5–5.4)
LDL/ApoB-100 ratio	1.0 (0.9–1.1)
LDL/HDL ratio	2.7 (1.8–3.5)
TG/HDL ratio	4.1 (2.9–5.3)
ApoB-100, mg/dL	90.0 (69.8–115.0)
Baseline Lp(a)	34.7 (14.8–79.0)
Glucose, mg/dL	111.0 (91.0–155.3)
HbA1c, %	6.0 (5.6–6.7)
Creatinine, mg/dL	0.9 (0.8–1.1)
eGFR, mL/min/1.73 m^2^	80.0 (67.5–90.0)
Urea, mg/dL	41.0 (35.0–50.0)
Uric acid, mg/dL	6.1 (4.8–7.5)
MACE	27 (11.5)

MI: myocardial infarction; PCI: percutaneous coronary intervention; STEMI: ST-elevation myocardial infarction; HDL-C: high-density lipoprotein cholesterol; LDL-C: low-density lipoprotein cholesterol; TG: triglycerides; VLDL: very-low-density lipoprotein; ApoB-100: apolipoprotein B-100; Lp(a): lipoprotein(a); HbA1c: hemoglobin A1c; eGFR: estimated glomerular filtration rate; MACE: major adverse cardiovascular events.

**Table 2:** Clinical characteristics of patients according to lipoprotein(a) variability. * Age-adjusted. Bold values indicate variables with *p* < 0.05.

Characteristic	High Lp(a) Variability (n = 136)	Low Lp(a) Variability (n = 99)	Adjusted *p*-Value
Age, years	61.4 (11.1)	61.9 (11.5)	0.720
Male sex	103 (75.7)	83 (83.8)	0.131
Diabetes mellitus	53 (39.0)	31 (31.6)	0.248
Hypertension	85 (62.5)	63 (63.6)	0.859
Dyslipidemia	104 (76.5)	70 (70.7)	0.320
Menopausal status *	**32 (23.5)**	**12 (12.1)**	**0.034**
Body mass index, kg/m^2^	28.1 (5.8)	27.8 (6.2)	0.795
Stroke	4 (2.9)	4 (4.0)	0.646
Transient ischemic attack	1 (0.7)	2 (2.0)	0.386
Peripheral artery disease	5 (3.7)	8 (8.1)	0.145
Prior MI	30 (22.1)	20 (20.2)	0.731
Prior PCI	25 (18.4)	18 (18.2)	0.969
Chronic kidney disease	69 (50.7)	49 (49.5)	0.851
Smoking status (never/former/current)	34/45/56	20/45/33	0.150
Alcohol consumption (never/former/current)	124/10/2	89/7/3	0.715
STEMI presentation	76 (55.9)	50 (50.5)	0.414
Number of treated lesions	2 (2)	2 (2)	0.628
Left main treated	8 (5.9)	7 (7.1)	0.713
Number of treated vessels	1.5 (1)	1 (1)	0.596
**Incomplete revascularization**	**43 (31.6)**	**18 (18.2)**	**0.020**
High-intensity statin	135 (99.3)	98 (99.0)	0.821
Ezetimibe	67 (49.3)	45 (45.5)	0.564
Total cholesterol, mg/dL	160.0 (61.0)	155.5 (73.0)	0.236
HDL-C, mg/dL	35.0 (11.0)	34.5 (9.0)	0.955
LDL-C, mg/dL	100.0 (57.0)	90.0 (64.0)	0.279
Non-HDL-C, mg/dL	128.0 (45.8)	120.3 (44.9)	0.203
Remnant-C, mg/dL	27 (13)	26 (12)	0.113
TG, mg/dL	147 (80)	134 (58)	0.087
VLDL, mg/dL	29.4 (15.9)	26.8 (11.6)	0.087
TC/HDL ratio	4.6 (2.2)	4.3 (1.4)	0.110
LDL/ApoB-100 ratio	0.98 (0.3)	0.99 (0.2)	0.501
LDL/HDL ratio	2.8 (1.9)	2.5 (1.3)	0.135
TG/HDL ratio	4.4 (2.4)	3.7 (2.5)	0.085
ApoB-100, mg/dL	94 (45)	86.5 (44)	0.078
Baseline Lp(a)	30.95 (51.4)	38.1 (65.1)	0.930
Follow-up Lp(a)	18.7 (64.0)	32.9 (61.7)	0.180
Glucose, mg/dL	116 (73)	109.5 (53)	0.302
HbA1c, %	5.9 (1.4)	6.0 (0.9)	0.895
Creatinine, mg/dL	0.96 (0.3)	0.95 (0.3)	0.969
eGFR, mL/min/1.73 m^2^	80 (23)	82 (22)	0.719
Urea, mg/dL	42 (18)	40 (14)	0.215
Uric acid, mg/dL	6.1 (2.9)	6.2 (2.9)	0.375
MACE	15 (11.0)	12 (12.1)	0.616

MI: myocardial infarction; PCI: percutaneous coronary intervention; STEMI: ST-elevation myocardial infarction; HDL-C: high-density lipoprotein cholesterol; LDL-C: low-density lipoprotein cholesterol; TG: triglycerides; VLDL: very-low-density lipoprotein; ApoB-100: apolipoprotein B-100; Lp(a): lipoprotein(a); HbA1c: hemoglobin A1c; eGFR: estimated glomerular filtration rate; MACE: major adverse cardiovascular events.

**Table 3:** Univariable and multivariable logistic regression analyses of independent predictors of high lipoprotein(a) variability. Variables with univariate *p*-value < 0.20 were considered for inclusion in multivariable logistic regression models and are shown in bold. * Age-adjusted.

	Univariable		Multivariable	
	OR (CI 95%)	*p*	OR (CI 95%)	*p*
Age, years	0.99 (0.97–1.02)	0.72	1.00 (0.98–1.03)	0.97
**Female sex**	**1.67 (0.85–3.22)**	**0.13**	**1.92 (0.93–4.00)**	**0.08**
Diabetes mellitus	1.38 (0.80–2.39)	0.25		
Hypertension	0.95 (0.56–1.63)	0.86		
Dyslipidemia	1.35 (0.75–2.42)	0.32		
Menopausal status *	**10.67 (1.10–105.30)**	**0.04**	**11.18 (0.79–157.58)**	**0.07**
Body mass index	1.01 (0.95–1.07)	0.87		
Stroke	0.72 (0.18–2.95)	0.65		
Transient ischemic attack	0.36 (0.03–4.12)	0.41		
**Peripheral artery disease**	**0.43 (0.14–1.37)**	**0.15**	**0.44 (0.12–1.61)**	**0.21**
Prior MI	1.12 (0.59–2.11)	0.73		
Prior PCI	1.01 (0.52–1.98)	0.97		
Chronic kidney disease	1.05 (0.63–1.76)	0.85		
Previous smoking	1.05 (0.75–1.48)	0.77		
Previous alcoholism	0.82 (0.42–1.62)	0.57		
STEMI presentation	1.24 (0.74–2.09)	0.41		
Number of treated lesions	1.06 (0.83–1.36)	0.64		
Left main treated	0.82 (0.29–2.35)	0.71		
Number of treated vessels	1.07 (0.78–1.49)	0.67		
**Incomplete revascularization**	**2.08 (1.11–3.89)**	**0.02**	**2.22 (1.14–4.31)**	**0.02**
Statin	1.38 (0.08–22.29)	0.82		
Ezetimibe	1.16 (0.69–1.96)	0.56		
Total cholesterol	1.00 (0.99–1.01)	0.22		
HDL-C	0.99 (0.97–1.03)	0.89		
LDL-C	1.00 (0.99–1.01)	0.35		
Non-HDL-C	1.00 (0.99–1.01)	0.20		
Remnant-C	1.01 (0.99–1.03)	0.31		
TG	1.00 (0.99–1.01)	0.21		
VLDL	1.01 (0.99–1.03)	0.21		
TC/HDL ratio	1.13 (0.94–1.36)	0.18		
LDL/ApoB-100 ratio	0.55 (0.17–1.73)	0.30		
**LDL/HDL ratio**	**1.18 (0.94–1.48)**	**0.15**	**0.98 (0.66–1.45)**	**0.98**
TG/HDL ratio	1.08 (0.97–1.20)	0.17		
**ApoB-100**	**1.01 (0.99–1.02)**	**0.07**	**1.01 (0.99–1.02)**	**0.25**
Baseline Lp(a)	1.00 (0.993–1.005)	0.80		
Follow-up Lp(a)	1.00 (0.994–1.004)	0.63		
**Glucose**	**1.00 (0.99–1.01)**	**0.15**	**1.00 (0.99–1.01)**	**0.36**
HbA1c	1.02 (0.83–1.24)	0.89		
Creatinine	1.11 (0.43–2.87)	0.82		
eGFR	0.99 (0.98–1.01)	0.72		
Urea	0.99 (0.99–1.01)	0.65		
Uric acid	1.05 (0.94–1.18)	0.38		
MACE	0.79 (0.33–2.95)	0.62		

OR: odds ratio; CI: confidence interval; MI: myocardial infarction; PCI: percutaneous coronary intervention; STEMI: ST-elevation myocardial infarction; HDL-C: high-density lipoprotein cholesterol; LDL-C: low-density lipoprotein cholesterol; TG: triglycerides; VLDL: very-low-density lipoprotein; ApoB-100: apolipoprotein B-100; Lp(a): lipoprotein(a); HbA1c: hemoglobin A1c; eGFR: estimated glomerular filtration rate; MACE: major adverse cardiovascular events.

## Data Availability

The data presented in this study are available on reasonable request from the corresponding author. The data are not publicly available due to privacy or ethical restrictions.

## Abbreviations

The following abbreviations are used in this manuscript:

Lp(a)	Lipoprotein(a)
LDL-C	Low-Density Lipoprotein Cholesterol
Apo(a)	Apolipoprotein(a)
ApoB-100	Apolipoprotein B-100
ASCVD	Atherosclerotic Cardiovascular Disease
ACS	Acute Coronary Syndrome
PCI	Percutaneous Coronary Intervention
MACE	Major Adverse Cardiovascular Events
MI	Myocardial Infarction
OR	Odds Ratio
CI	Confidence Interval
IQR	Interquartile Range
hsCRP	High-Sensitivity C-Reactive Protein
HR	Hazard Ratio
CAD	Coronary Artery Disease

## Appendix A

### Inclusion and Exclusion Criteria

Inclusion criteria in Cohort A were ACS patients in whom at least two of the following criteria were present: age > 65 years, diabetes mellitus, multivessel CAD, peripheral artery disease, chronic kidney disease, prior stroke (anytime) or prior transient ischemic attack in the last 6 months, prior MI, complex PCI, prior PCI or coronary artery bypass grafting, heart failure, body mass index > 27 kg/m^2^, anticipated long-term use of an oral anticoagulant, hemoglobin < 11 g/dL, spontaneous bleeding requiring hospitalization or transfusion in the past 12 months, bleeding diathesis, active malignancy, or previous spontaneous intracranial hemorrhage.

Inclusion criteria in Cohort B were patients aged <75 years who were admitted for ACS between 2019 and 2020.

Exclusion criteria were the same in both cohorts and comprised age < 18 years, low life expectancy (<1 year) in the judgement of the treating physician, pregnant or breastfeeding women, and evidence at coronary angiography of non-significant coronary artery disease (<30% stenosis in the left main stem or <50% in other coronary segments).

## Appendix B

**Table A1:** Baseline characteristics of Cohort A and Cohort B. Continuous variables are reported as mean ± standard deviation when normally distributed and as median (interquartile range) when non-normally distributed. Categorical variables are reported as n (%). Significant imbalances among groups are highlighted in bold.

	Cohort A (n = 143)	Cohort B (n = 92)
Baseline Lp(a)	36.5 (15.6–83.1)	30.6 (11.3–62.4)
**Age, years**	**67.0 (58.4–74.9)**	**56.0 (52.0–60.0)**
Male sex	111 (77.6)	75 (81.5)
**Diabetes mellitus**	**62 (43.7)**	**22 (23.9)**
**Hypertension**	**99 (69.2)**	**49 (53.3)**
Dyslipidemia	108 (75.5)	66 (71.7)
Menopausal status	31 (32)	13 (17)
Body mass index, kg/m^2^	27.6 (25.3–30.9)	28.4 (25.5–31.8)
Stroke	7 (4.9)	1 (1.1)
Transient ischemic attack	2 (1.4)	1 (1.1)
**Peripheral artery disease**	**12 (8.4)**	**1 (1.1)**
**Prior MI**	**40 (28.0)**	**10 (10.9)**
**Prior PCI**	**39 (27.3)**	**4 (4.3)**
**Chronic kidney disease**	**106 (74.1)**	**12 (13.0)**
Smoking status (former or current)	103 (73)	76 (82.6)
Alcohol consumption (former or current)	14 (9.8)	8 (8.7)
STEMI presentation	76 (53.1)	50 (54.3)
Number of treated lesions	2.0 (1.1)	1.8 (1.0)
Left main treated	12 (8.4)	3 (3.3)
Number of treated vessels	1.7 (0.8)	1.7 (0.9)
**Incomplete revascularization**	**24 (16.8)**	**37 (40.2)**
High-intensity statin	141 (98.6)	92 (100)
**Ezetimibe**	**85 (59.4)**	**27 (29.3)**
**Total cholesterol, mg/dL**	**145.0 (113.5–179.0)**	**180.0 (150.5–211.5)**
**HDL-C, mg/dL**	**34.0 (31.0–39.0)**	**37.5 (32.0–42.5)**
**LDL-C, mg/dL**	**83.0 (55.5–114.5)**	**112.5 (89.5–138.0)**
**Non–HDL-C, mg/dL**	**109.0 (83.0–143.0)**	**142.5 (114.0–164.5)**
**Remnant-C, mg/dL**	**25.0 (20.0–33.0)**	**28.0 (22.0–37.0)**
TG, mg/dL	140.0 (110.0–185.5)	139.0 (108.0–178.0)
VLDL, mg/dL	28.0 (22.0–37.1)	27.8 (21.6–35.6)
**TC/HDL ratio**	**4.3 (3.3–5.1)**	**4.7 (4.2–5.5)**
**LDL/ApoB-100 ratio**	**1.0 (0.8–1.1)**	**1.1 (0.9–1.2)**
**LDL/HDL ratio**	**2.4 (1.6–3.2)**	**2.9 (2.3–3.6)**
TG/HDL ratio	4.3 (2.9–5.5)	3.7 (2.9–4.9)
**ApoB-100, mg/dL**	**83.0 (67.0–111.5)**	**103.5 (83.0–123.0)**
Follow-up Lp(a)	31.8 (10.0–75.0)	18.2 (5.4–62.1)
Glucose, mg/dL	111.0 (91.5–155.0)	111.5 (91.0–154.5)
**HbA1c, %**	**6.0 (5.6–6.8)**	**5.9 (5.5–6.3)**
**Creatinine, mg/dL**	**1.0 (0.9–1.2)**	**0.9 (0.8–1.0)**
**eGFR, mL/min/1.73 m^2^**	**75.0 (58.5–85.0)**	**89.0 (80.0–90.0)**
**Urea, mg/dL**	**43.0 (37.0–56.0)**	**36.5 (31.5–45.0)**
Uric acid, mg/dL	5.9 (4.8–7.4)	6.6 (5.2–7.8)
**MACE**	**7 (4.9)**	**20 (21.7)**

MI: myocardial infarction; PCI: percutaneous coronary intervention; STEMI: ST-elevation myocardial infarction; HDL-C: high-density lipoprotein cholesterol; LDL-C: low-density lipoprotein cholesterol; TG: triglycerides; VLDL: very-low-density lipoprotein; ApoB-100: apolipoprotein B-100; Lp(a): lipoprotein(a); HbA1c: hemoglobin A1c; eGFR: estimated glomerular filtration rate; MACE: major adverse cardiovascular events.

**Table A2:** Patient characteristics according to baseline and follow-up lipoprotein(a) risk category. Bold values indicate variables with *p* < 0.05.

Baseline Lp(a) Risk Category	Normal (<30 mg/dL)			Intermediate (30–49 mg/dL)			High (≥50 mg/dL)			*p*-Value
N (%)	109 (46.4)			41 (17.4)			85 (36.2)			
Follow-Up Lp(a) Risk Category	Normal	Intermediate	High	Normal	Intermediate	High	Normal	Intermediate	High	
N (%)	103 (94.5)	6 (5.5)	0	17 (41.5)	16 (39.0)	8 (19.5)	4 (4.7)	9 (10.6)	72 (84.7)	
Age, years, median (IQR)	59.8 (12.8)	68.0 (12.4)	-	64.5 (15.9)	61.6 (20.9)	62.0 (9.3)	56.5 (10.8)	67.4 (16.9)	61.0 (22.9)	0.59
Male sex, n (%)	82 (79.6)	4 (66.7)	-	14 (82.4)	15 (93.8)	6 (75.0)	3 (75.0)	6 (66.7)	56 (77.8)	0.80
Diabetes mellitus, n (%)	36 (35.0)	2 (33.3)	-	10 (58.8)	7 (46.7)	3 (37.5)	0	4 (44.4)	22 (30.6)	0.32
Hypertension, n (%)	59 (57.3)	5 (83.3)	-	11 (64.7)	10 (62.5)	6 (75.0)	1 (25.0)	5 (55.6)	51 (70.8)	0.37
Dyslipidemia, n (%)	73 (70.9)	5 (83.3)	-	11 (64.8)	11 (68.8)	8 (100)	3 (75.0)	7 (77.8)	56 (77.8)	0.64
Menopausal status, n (%)	20 (95.2)	2 (100)	-	3 (100)	1 (100)	2 (100)	1 (100)	3 (100)	12 (75.0)	0.56
Body mass index, kg/m^2^, median (IQR)	28.1 (5.6)	29.1 (8.7)	-	28.4 (6.2)	28.1 (4.2)	29.2 (5.9)	25.5 (3.6)	26.7 (8.0)	27.6 (7.2)	0.74
Stroke, n (%)	0	0	-	1 (5.9)	1 (6.3)	1 (12.5)	0	0	5 (6.9)	0.20
Transient ischemic attack, n (%)	1 (1.0)	0	-	1 (5.9)	0	0	0	0	1 (1.4)	0.84
Peripheral artery disease, n (%)	4 (3.9)	0	-	0	0	0	0	0	8 (11.1)	0.30
Prior MI, n (%)	22 (21.4)	1 (16.7)	-	3 (17.6)	3 (18.8)	2 (25.0)	1 (25.0)	2 (22.2)	16 (22.2)	0.99
Prior PCI, n (%)	19 (18.4)	0	-	3 (17.6)	2 (12.5)	2 (25.0)	1 (25.0)	2 (22.2)	14 (19.4)	0.95
Chronic kidney disease, n (%)	50 (48.5)	5 (83.3)	-	5 (29.4)	6 (37.5)	5 (62.5)	1 (25.0)	6 (66.7)	40 (55.6)	0.19
Previous smoking, n (%)	79 (77.5)	3 (50.0)	-	14 (82.4)	14 (93.3)	6 (75.0)	4 (100)	8 (88.9)	51 (70.8)	0.30
Previous alcoholism, n (%)	12 (11.7)	0	-	3 (17.6)	0	1 (12.5)	0	1 (11.1)	5 (6.9)	0.60
STEMI, n (%)	55 (53.4)	2 (33.3)	-	14 (82.4)	8 (50.0)	6 (75.0)	2 (50.0)	4 (44.4)	35 (48.6)	0.24
Number of treated lesions, mean (SD)	1.83 (1.0)	1.83 (1.0)	-	1.88 (0.9)	2.13 (1.3)	3.13 (1.6)	2.0 (1.2)	1.67 (0.7)	2.03 (1.0)	0.08
Left main treated, n (%)	3 (2.9)	0	-	1 (5.9)	1 (6.3)	1 (12.5)	0	1 (11.1)	8 (11.1)	0.51
Number of treated vessels, mean (SD)	1.61 (0.8)	1.5 (0.6)	-	1.65 (0.9)	1.63 (0.9)	2.25 (0.9)	1.5 (1.0)	1.56 (0.5)	1.75 (0.8)	0.52
**Incomplete revascularization, n (%)**	**26 (25.2)**	**2 (33.3)**	-	**4 (23.5)**	**1 (6.3)**	**6 (75.0)**	**4 (100.0)**	**2 (22.2)**	**16 (22.2)**	**<0.001**
Statin, n (%)	102 (99.0)	6 (100.0)	-	17 (100.0)	16 (100.0)	8 (100.0)	4 (100.0)	8 (88.9)	72 (100.0)	0.09
Ezetimibe, n (%)	42 (40.8)	3 (50.0)	-	8 (47.1)	10 (62.5)	4 (50.0)	2 (50.0)	4 (44.4)	39 (54.2)	0.70
Total cholesterol, median (IQR)	156.0 (57.0)	166.5 (62.0)	-	133.0 (38.0)	160 (108.0)	179.0 (50.0)	207.5 (27.0)	137.5 (90.0)	159 (82.0)	0.58
HDL-C, median (IQR)	34 (7.0)	41 (11.0)	-	31.5 (12.0)	33 (13.0)	32.5 (7.0)	39.0 (11.0)	32.0 (16.0)	36.0 (12.0)	0.24
LDL-C, median (IQR)	97 (58.0)	105.5 (66.0)	-	74.5 (44.0)	91.0 (93.0)	113.0 (40.0)	134.5 (14.0)	81.0 (84.0)	92.5 (60.0)	0.54
Non-HDL-C, median (IQR)	122.0 (57.0)	130.0 (66.0)	-	98.0 (54.0)	125.0 (101.0)	141.0 (54.0)	164.5 (24.0)	109.5 (85.0)	118.5 (71.0)	0.51
Remnant-C, median (IQR)	25 (12.0)	31.5 (12.0)	-	23.5 (8.0)	30.0 (15.0)	26.5 (57.0)	26.0 (17.0)	27.0 (17.0)	27.0 (14.0)	0.99
TG, median (IQR)	141.0 (78.0)	118.0 (115.0)	-	118.5 (37)	139.0 (81.0)	153.5 (262.0)	146.5 (54.0)	133.5 (43.0)	146.0 (83.0)	0.84
VLDL, median (IQR)	28.2 (15.6)	23.6 (22.9)	-	23.7 (7.4)	27.8 (16.2)	30.7 (52.4)	29.3 (10.8)	26.7 (8.6)	29.2 (16.5)	0.84
TC/HDL ratio, median (IQR)	4.4 (0.9)	4.2 (1.8)	-	4.0 (1.5)	4.6 (2.8)	4.8 (2.5)	5.3 (0.7)	4.5 (2.7)	4.3 (1.7)	0.46
LDL/ApoB-100 ratio, median (IQR)	1.0 (0.2)	1.1 (0.4)	-	0.9 (0.1)	1.0 (0.3)	1.0 (0.3)	1.1 (0.2)	0.9 (0.2)	1.0 (0.3)	0.31
LDL/HDL ratio, median (IQR)	2.6 (1.7)	2.7 (1.7)	-	2.2 (1.1)	2.8 (2.7)	3.1 (1.5)	3.5 (0.8)	2.5 (2.8)	2.6 (1.4)	0.64
TG/HDL ratio, median (IQR)	3.9 (2.7)	3.2 (3.7)	-	3.2 (2.1)	3.7 (2.3)	4.7 (9.4)	4.2 (2.0)	4.4 (2.4)	4.3 (2.1)	0.82
ApoB-100, median (IQR)	87.0 (42.0)	88.0 (38.0)	-	75.0 (37.0)	98.0 (51.0)	114.5 (33.0)	120.0 (25.0)	85.0 (58.0)	90.5 (49.0)	0.45
**Baseline Lp(a), median (IQR)**	**12.3 (13.8)**	**23.4 (3.9)**	**-**	**36.6 (9.9)**	**39.7 (6.8)**	**39.4 (6.4)**	**60.4 (33.6)**	**60.4 (28.4)**	**102.5 (44.4)**	**<0.001**
**Follow-up Lp(a), median (IQR)**	**8.7 (10.5)**	**36.5 (4.4)**	**-**	**16.3 (12.1)**	**42.4 (13.3)**	**61.8 (28.1)**	**19.7 (15.3)**	**37.7 (13.8)**	**105.5 (55.3)**	**<0.001**
Glucose, median (IQR)	115.0 (64.0)	100.0 (37.0)	-	129.0 (67.0)	110.0 (74.0)	159.0 (87.0)	88.5 (69.0)	101.0 (124.0)	111.0 (58)	0.78
HbA1c, median (IQR)	6.0 (1.3)	5.7 (0.9)	-	6.6 (2.2)	6.3 (0.7)	5.9 (1.5)	5.7 (0.7)	6.2 (1.5)	5.9 (0.9)	0.45
Creatinine, median (IQR)	0.91 (0.3)	0.98 (0.3)	-	0.98 (0.3)	0.85 (0.3)	1.0 (0.5)	0.9 (0.2)	1.0 (0.3)	1.0 (0.4)	0.10
**eGFR, median (IQR)**	**82.0 (17.0)**	**77.0 (16.0)**	**-**	**78.5 (25.0)**	**90.0 (18.0)**	**78.5 (29.0)**	**90.0 (11.0)**	**77.0 (30.0)**	**77.0 (33.0)**	**0.02**
Urea, median (IQR)	41.0 (13.0)	36.0 (11.0)	-	40.5 (22.0)	36.0 (13)	42.0 (43.0)	43.5 (32.0)	47.5 (26.0)	43.5 (15)	0.15
Uric acid, median (IQR)	6.3 (2.9)	6.3 (1.9)	-	5.7 (2.8)	4.8 (2.7)	7.5 (2.7)	4.6 (5.4)	5.7 (2.4)	6.3 (2.5)	0.14
MACE, n (%)	8 (18.2)	0	-	4 (33.3)	2 (33.3)	0	0	0	13 (46.4)	0.15
**High Lp(a) variability, n (%)**	**59 (57.3)**	**6 (100.0)**	**-**	**15 (88.2)**	**1 (6.3)**	**8 (100.0)**	**4 (100.0)**	**7 (77.8)**	**36 (50.0)**	**<0.01**

Lp(a): lipoprotein(a); MI: myocardial infarction; PCI: percutaneous coronary intervention; STEMI: ST-elevation myocardial infarction; HDL-C: high-density lipoprotein cholesterol; LDL-C: low-density lipoprotein cholesterol; TG: triglycerides; VLDL: very-low-density lipoprotein; ApoB-100: apolipoprotein B-100; HbA1c: hemoglobin A1c; eGFR: estimated glomerular filtration rate; MACE: major adverse cardiovascular events.

**Table A3:** Reclassification of patients across lipoprotein(a) risk categories and intra-individual variability.

Baseline Lp(a) Risk Category	Follow-Up Lp(a) Risk, N (%)	Lp(a) Variability	N (%)
Low-risk (109), Low	Low-risk, 103 (94.5)	High	59 (57.3)
variability 50 (45.9%), High		Low	44 (42.7)
variability 59 (54.1%)	Intermediate-risk, 6 (5.5)	High	0 (0.0)
		Low	6 (100.0)
	High-risk, 0 (0%)	High	0 (0.0)
		Low	0 (0.0)
Intermediate-risk (41), Low	Low-risk, 17 (41.5)	High	15 (88.2)
variability 17 (41.5%), High		Low	2 (11.8)
variability 24 (58.5%)	Intermediate-risk, 16 (39)	High	1 (6.3)
		Low	15 (93.7)
	High-risk, 8 (19.1)	High	8 (100)
		Low	0 (0.0)
High-risk (85), Low	Low-risk, 4 (4.7)	High	4 (100.0)
variability 38 (44.7%), High		Low	0 (0.0)
variability 47 (55.3%)	Intermediate-risk, 9 (10.6)	High	7 (77.8)
		Low	2 (22.2)
	High-risk, 72 (84.7)	High	36 (50.0)
		Low	36 (50.0)
